# Hybrid Argon Plasma Coagulation for Treatment of Gastric Intestinal Metaplasia

**DOI:** 10.7759/cureus.7427

**Published:** 2020-03-26

**Authors:** Elias Estifan, Yana Cavanagh, Matthew A Grossman

**Affiliations:** 1 Internal Medicine, St. Joseph's University Medical Center, Paterson, USA; 2 Interventional Gastroenterology, St. Joseph's University Medical Center, Paterson, USA

**Keywords:** hybrid argon plasma coagulation, gastric intestinal metaplasia, endoscopic submucosal dissection

## Abstract

Hybrid argon plasma coagulation (HybridAPC® [HAPC]) is an evolution of the standard argon plasma coagulation (APC) technology, where the application of APC is preceded by high-pressure needleless submucosal injection. APC is indicated for the ablation of benign and dysplastic mucosal lesions, such as vascular malformations or Barrett's mucosa. HAPC offers safety and efficacy advantages over standard APC because the submucosal injection acts as a heat sink that disperses energy. This ensures that the underlying muscularis propria remains unaffected, and only the mucosal layer is coagulated in its entirety. An 81-year-old Hispanic male was found to have a 1.2-cm mucosal nodule along the incisura of the stomach. Pathology of the biopsy specimen revealed high-grade dysplasia, and he subsequently underwent endoscopic ultrasound examination, which confirmed the presence of an isolated gastric nodule with no deep invasion of the muscularis propria, consistent with a uT1N0Mx endosonographic staging. He then underwent endoscopic submucosal dissection of the lesion. Pathology of the excised specimen confirmed the presence of multifocal high-grade dysplasia, arising in the background of extensive intestinal metaplasia. The deep margin was clear; however, the lateral resection margins showed focal involvement of intestinal metaplasia with low-grade dysplasia. Surveillance endoscopy confirmed the persistence of diffuse intestinal metaplasia. He was then treated with widespread HAPC due to the presence of underlying diffuse intestinal metaplasia in the stomach. HAPC is an effective and efficient treatment modality for mucosal lesions. In one series of 50 patients, 96% achieved complete macroscopic remission of Barrett's mucosa after a median of 3.5 APC sessions, and 85% achieved complete histological remission. HAPC is a promising therapeutic modality as a thermal injury is targeted, and the depth of injury is contained. This provides immediate procedural efficacy and safety benefits, and reduces subsequent complications when compared with standard APC. We anticipate that the applications of HAPC will continue to grow, as this modality is adopted into common procedural parlance. This case appears to be the first to describe the use of HAPC for definitive treatment of diffuse intestinal metaplasia.

## Introduction

Gastric cancer (GC) is the fifth most common cancer worldwide [1]. Its incidence is highest in China, Japan, South Korea, and East Europe, with a lower incidence in North America, Western Europe, and South-Central Asia [1]. In Japan, the five-year survival rate reaches up to 90% due to early detection from established screening protocols [2]. However, in Europe, the survival rates are only 10%-30% as there are is no formal guidelines for GC screening, and patients often do not seek medical attention until advanced stages of the disease when symptoms develop [2]. The Lauren classification is a system for differentiation of GC into diffuse and intestinal subtypes [3]. Notably, despite the divergence in prognosis, there is no difference in the clinical management of the two histological types [4]. Perioperative chemotherapy and chemoradiation may improve outcomes of resectable lesions. However, prognosis remains poor, with five-year survival rates of 20%-30% in patients with positive node(s). Surgery is still considered the only curative approach to GC, although endoscopic resections for early-stage lesions appear promising as a therapeutic modality [5].

Correa et al. described a stepwise progression that ultimately results in the formation of GC [6]. The initial step of the cascade appears to be the evolution of chronic gastritis, which later progresses to atrophic gastritis, intestinal metaplasia, dysplasia, and then gastric cancer [7]. Interestingly, Helicobacter pylori is one of the leading causes of chronic gastritis, and its eradication can lead to a regression of the histological features of chronic gastritis [7]. Although there is no guarantee or timeline for the development of GC, the progression to intestinal metaplasia is the point of no return [8]. The presence of background intestinal metaplasia allows for the evolution of low-grade dysplasia (due to early APC gene mutations) and high-grade dysplasia (due to TP53 mutations) which are associated with a higher risk of ultimate progression to GC [1].

Endoscopic submucosal dissection (ESD) was initially performed in 1988 for the treatment of early superficial gastric cancer [9]. Over the last few decades, ESD has evolved and is now implemented in the management of large mucosal and submucosal lesions [10]. ESD is currently indicated for the treatment of esophageal cancer, including intramucosal neoplasms limited to the lamina propria and occupying <2/3 of the lumen of the esophagus with no lymph node involvement. Relative indications can include resection of deeper lesions involving less than 200 µm of the submucosa [11]. In early GC, ESD can be considered in well-differentiated T1a adenocarcinoma with a diameter less than 2 cm, and no lymph node infiltration [12].

APC is a flexible probe insert through the working channel of the endoscope and extending to a visible length of 10 mm outside the scope. This catheter carries out thermal ablation via an argon gas flow conduit [13]. Hybrid argon plasma coagulation (HybridAPC® [HAPC]) is a novel ablation technique where the application of APC is preceded by a high-pressure needleless submucosal injection of saline. This is accomplished via a built-in water jet within the same catheter as the APC channel [14]. HAPC is indicated for the management of Barrett’s esophagus; however, we are describing a novel use of HAPC in the treatment of gastric intestinal metaplasia with dysplasia [15].

## Case presentation

**Figure 1:**
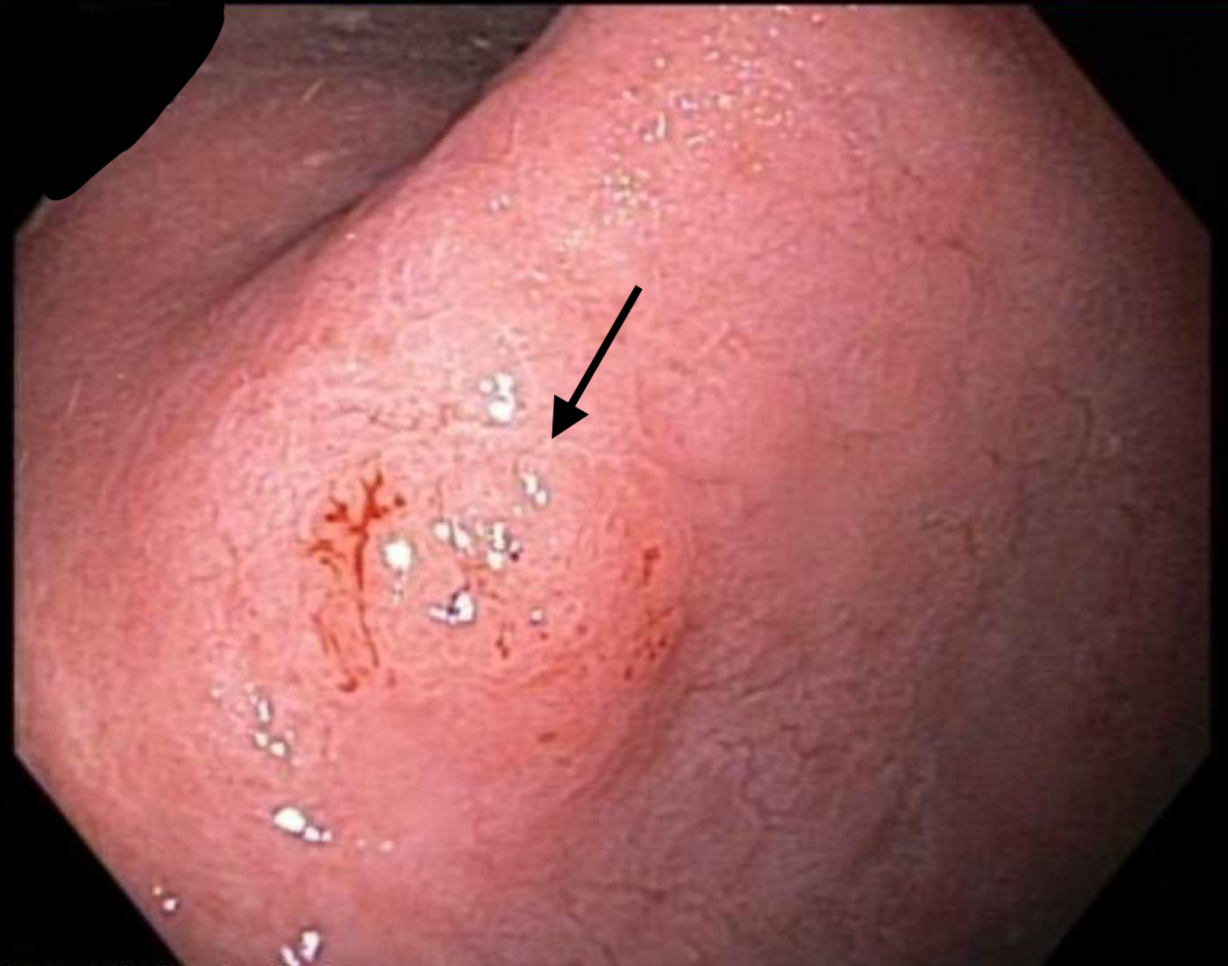
Endoscopic image Endoscopic view of the incisura of the stomach revealing a 1.2-cm mucosal nodule.

An 81-year-old Hispanic male was found to have a 1.2-cm mucosal nodule along the incisura of the stomach (Figure 1).

**Figure 2:**
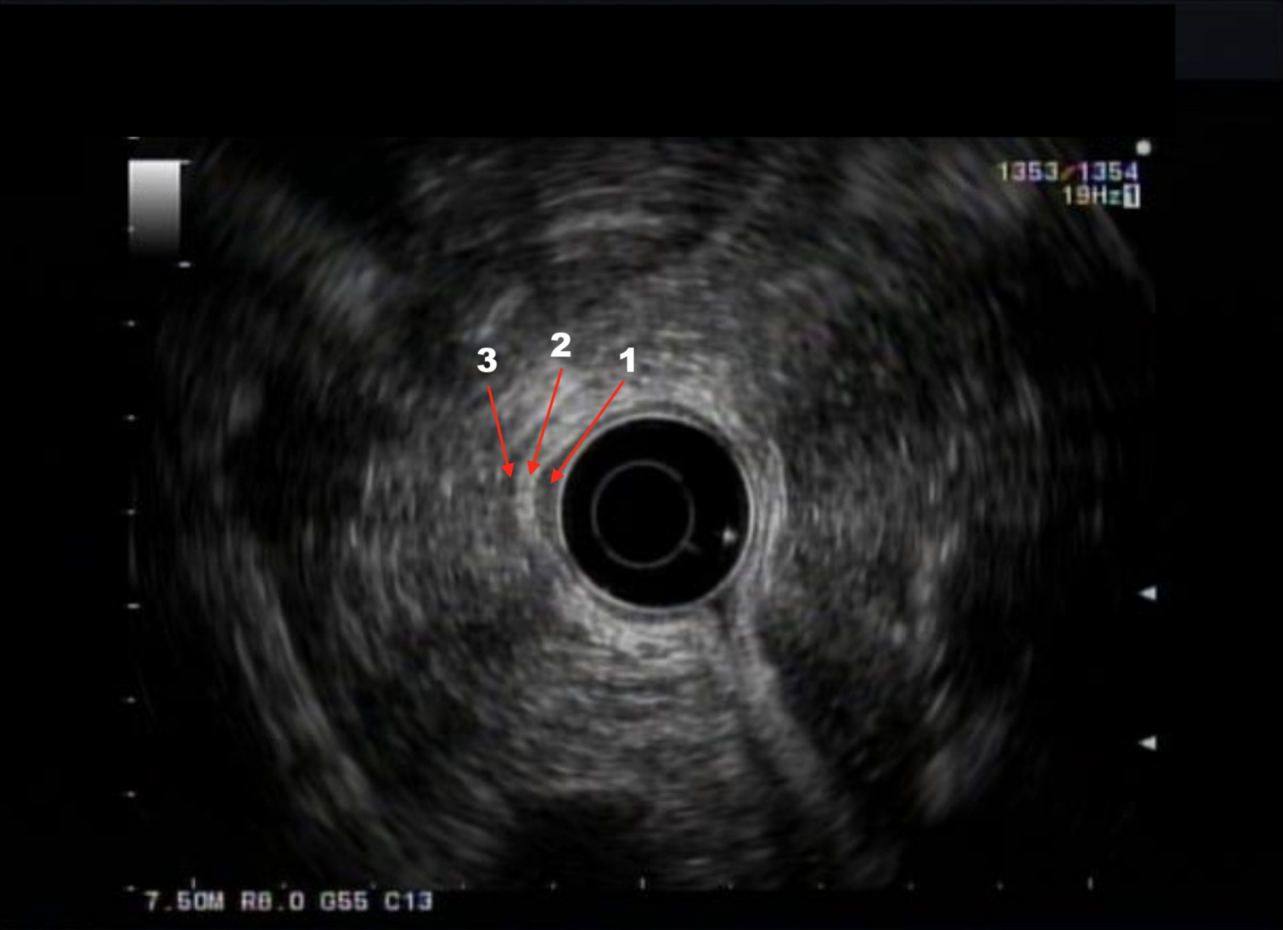
Endoscopic ultrasound (EUS) imaging EUS showing isolated mucosal nodule (arrow number 1) with no deep invasion of the submucosa (arrow number 2) or muscularis propria (arrow number3)

Pathology of the biopsy specimen revealed high-grade dysplasia in the setting of intestinal metaplasia, and he subsequently underwent endoscopic ultrasound (EUS) examination. EUS confirmed the presence of an isolated mucosal nodule with no deep invasion into the submucosa or muscularis propria (Figure 2).

**Figure 3:**
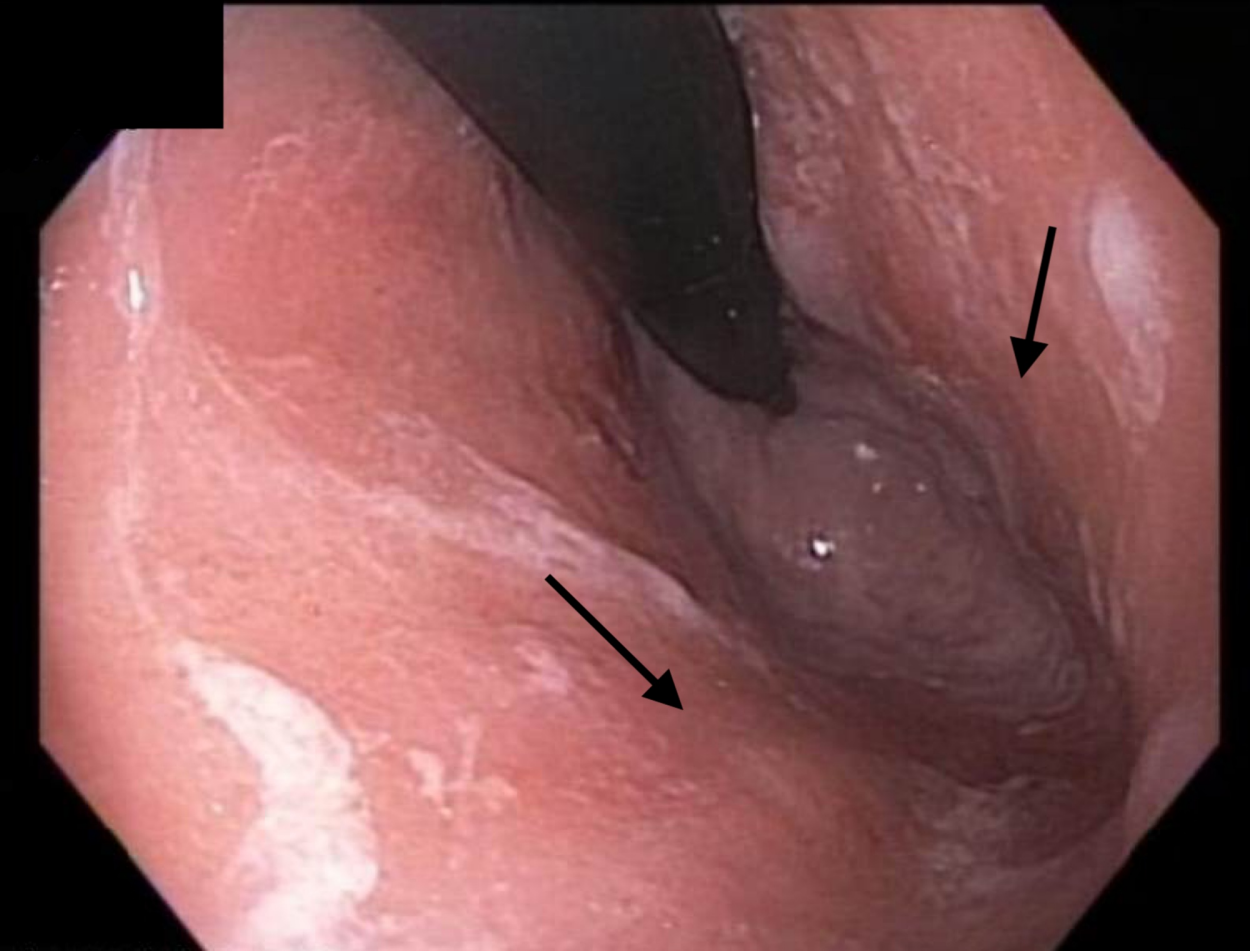
Endoscopic image Endoscopic view of the gastric body and fundus revealing normal gastric mucosa.

The decision was made to resect the lesion en bloc using ESD (Figure 3). 

**Figure 4:**
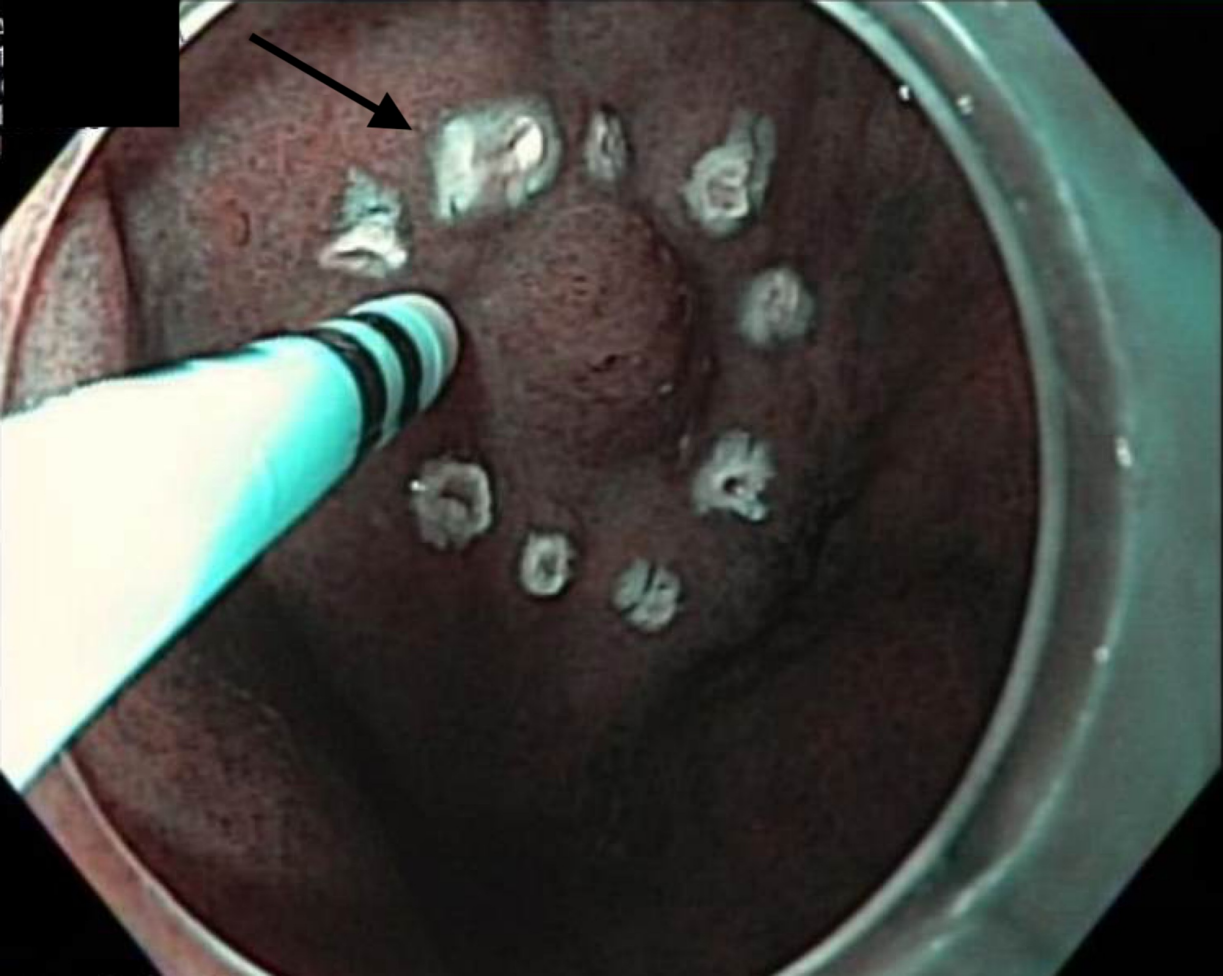
Endoscopic image Endoscopic view of the aforementioned mucosal nodule under narrow-band imaging (NBI) demonstrating the demarcated margins of the anticipated endoscopic submucosal dissection (ESD) field.

A standard gastroscope was fitted with a distal attachment (cap) and introduced to identify the area of interest, along the lesser curvature of the stomach. An ERBE I-type Hybridknife was used to demarcate the peripheral margins of the lesion in a circular fashion (Figure 4).

**Figure 5:**
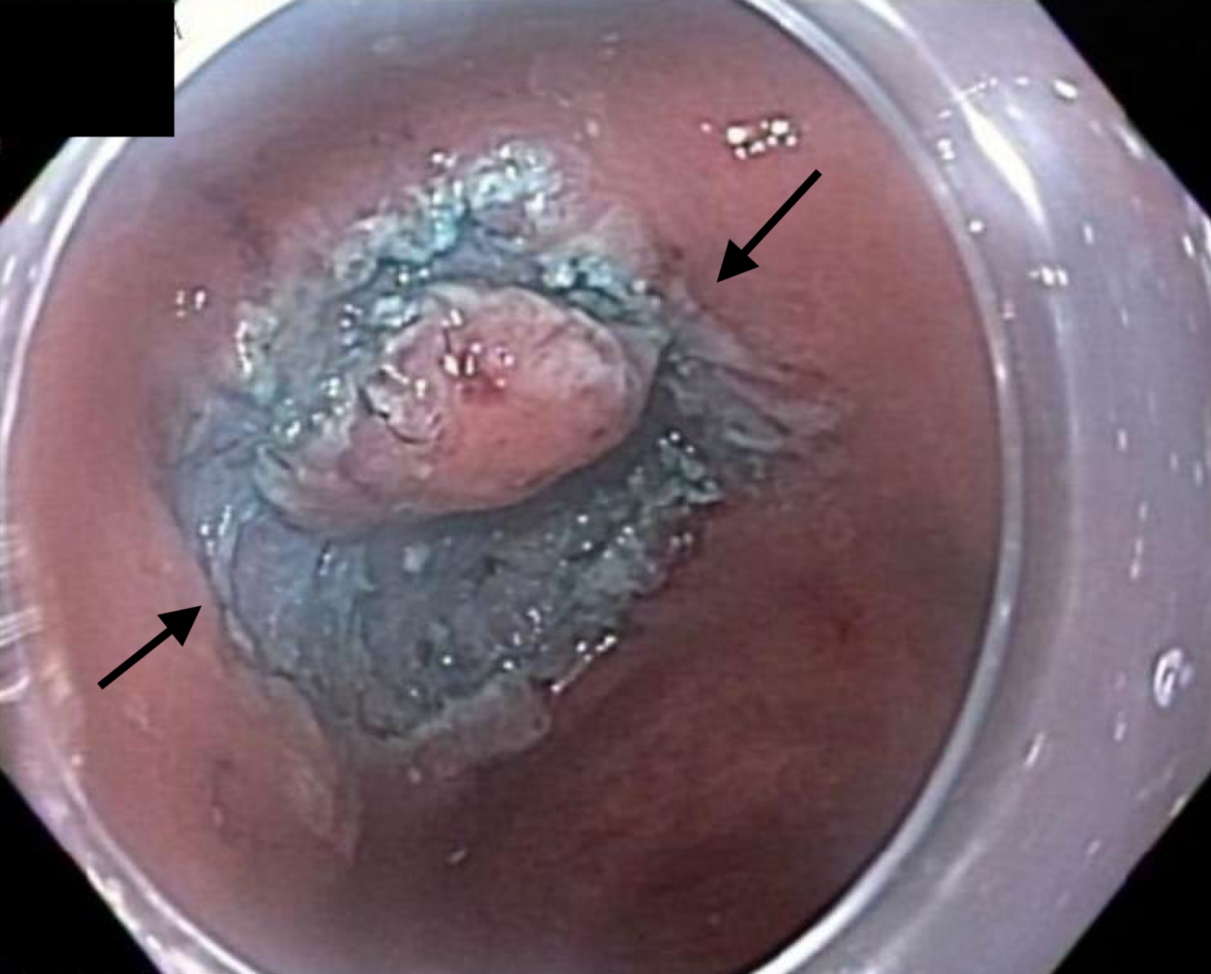
Endoscopic image Endoscopic view of the aforementioned mucosal nodule following injection of a mixture of saline and methylene blue and subsequent circumferential incision of the demarcated margins of the endoscopic submucosal dissection (ESD) field.

 The ERBE I-type Hybridknife was then used to inject a mixture of saline and methylene blue into the submucosal plane (Figure 5).

**Figure 6:**
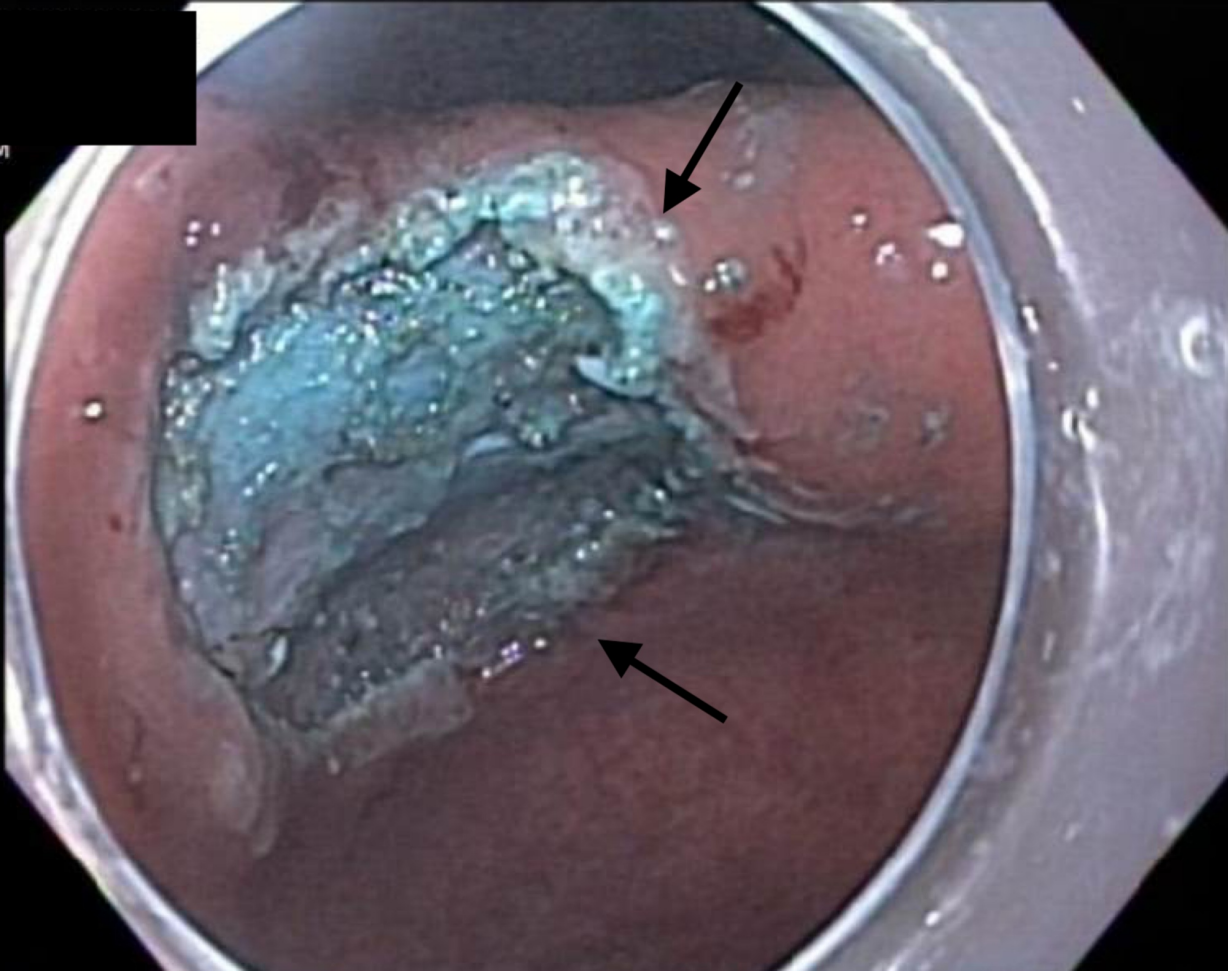
Endoscopic image Endoscopic appearance of the incisura following completion of the endoscopic submucosal dissection (ESD).

The lesion lifted symmetrically, and an en bloc ESD was successfully performed (Figure 6). 

**Figure 7:**
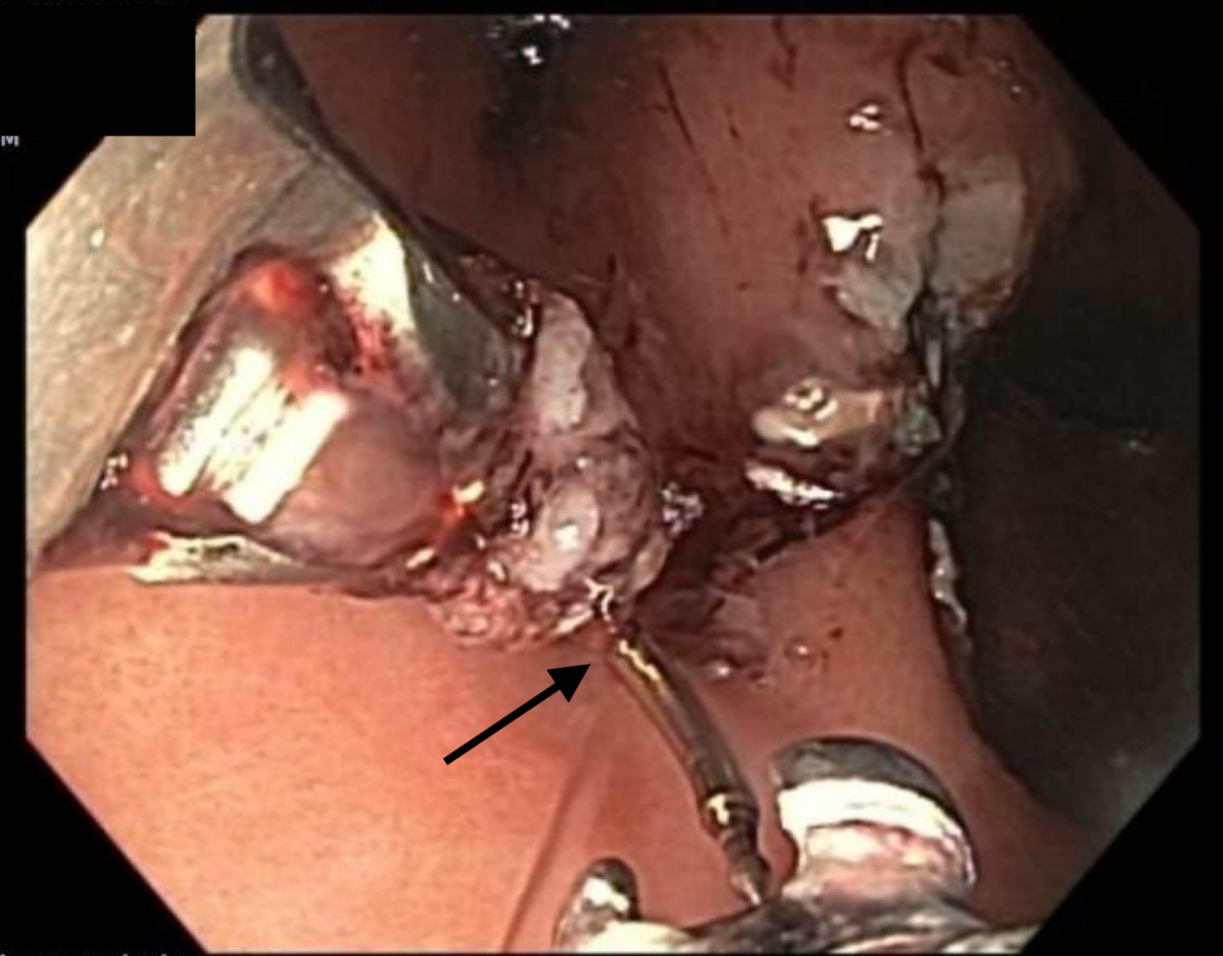
Endoscopic image Endoscopic suturing for the closure of the mucosal defect following completion of endoscopic submucosal dissection (ESD) of the mucosal nodule.

On completion of the ESD, the residual mucosal defect was approximately 3 cm in diameter. The mucosal defect was closed with a running endoscopic suture (Figure 7).

**Figure 8:**
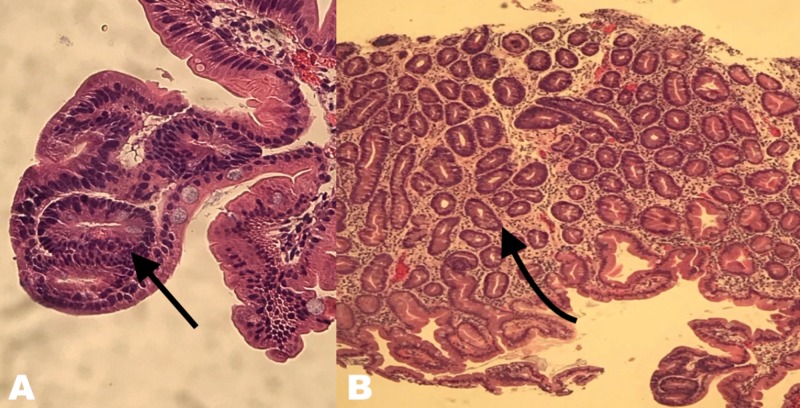
Pathology image (A) Pathology image of high magnification of hematoxylin and eosin (H&E) stain revealing multifocal high-grade dysplasia arising in the background of extensive intestinal metaplasia. (B) Pathology image of low magnification of H&E stain.

Pathology of the specimen showed multifocal high-grade dysplasia arising in the background of extensive intestinal metaplasia (Figure 8). The resection margins were focally involved by intestinal metaplasia with low-grade dysplasia, and the deep resection margin was unremarkable. 

**Figure 9:**
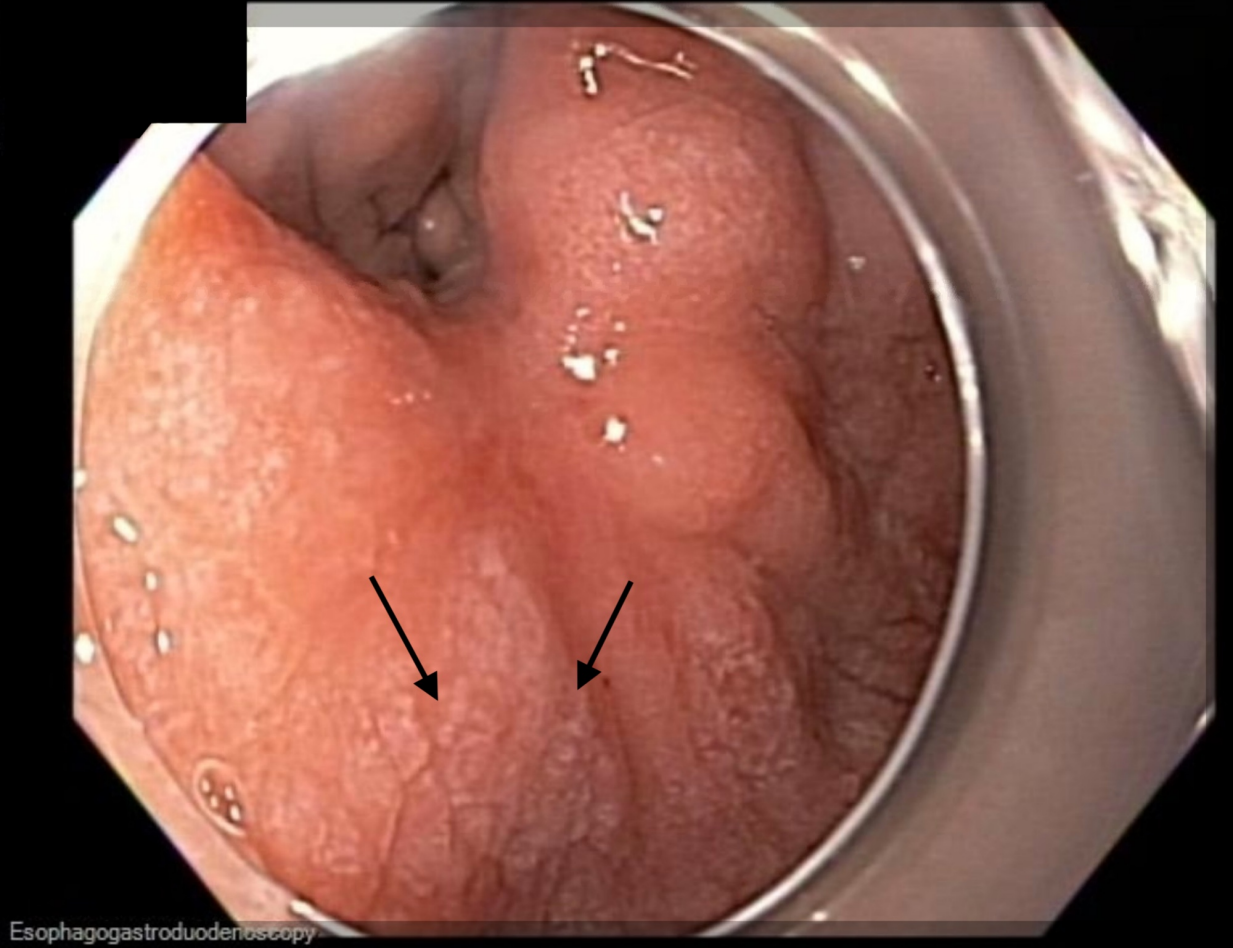
Endoscopic image Endoscopic image showing residual intestinal metaplasia with dysplasia and distal body and antrum displayed diffuse irregular whitish mucosa, similar to the appearance of diffuse lymphangiectasias.

In light of the persistent residual intestinal metaplasia with low-grade dysplasia, the patient returned for HAPC of the residual metaplastic mucosa (Figure 9).

**Figure 10:**
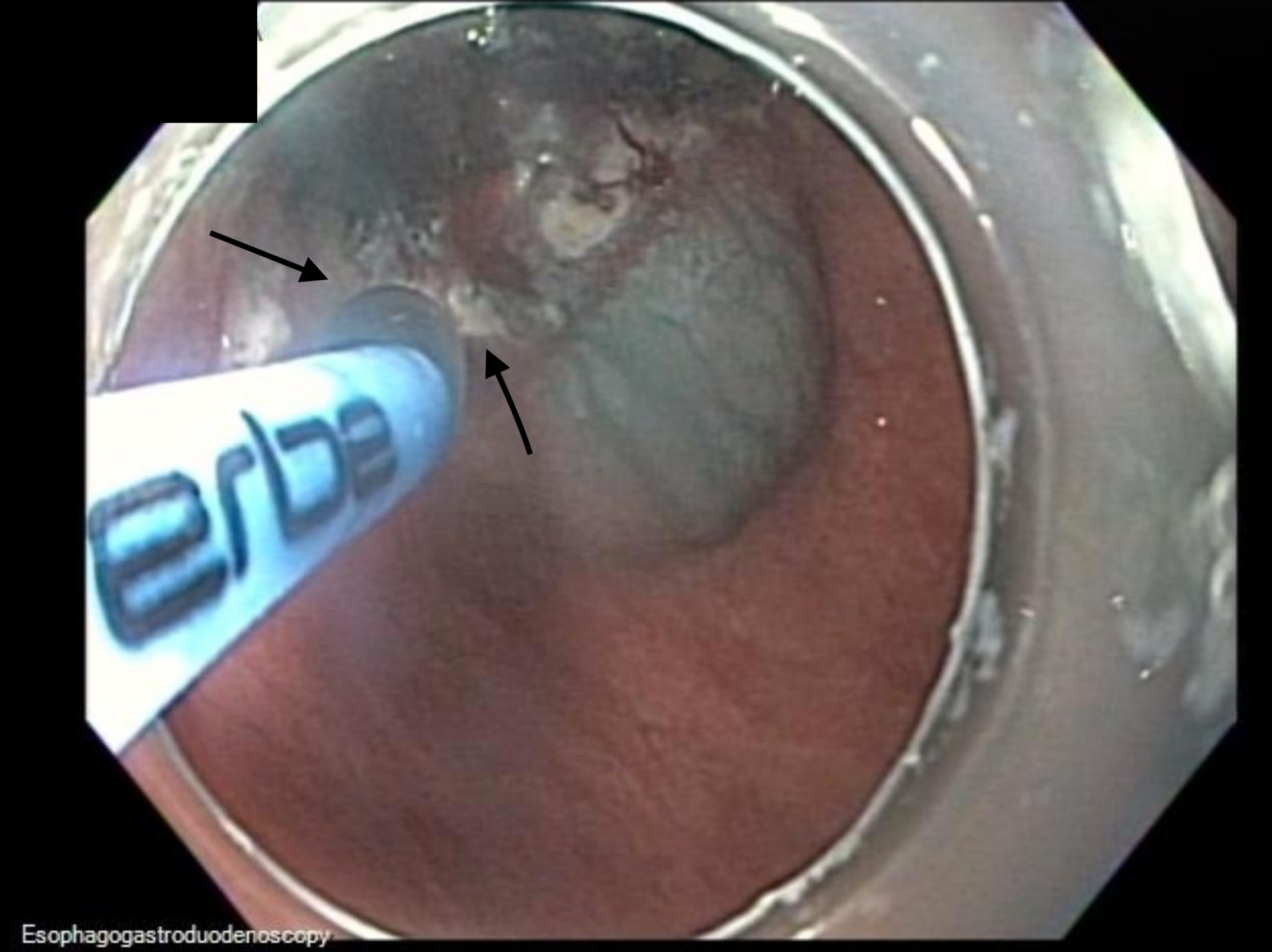
Endoscopic image Endoscopic image showing lifting the gastric mucosa with a solution of saline and methylene blue and ablated using argon plasma coagulation at a power setting of 50 W in a circumferential manner.

The scar site of the ESD was identified at the incisura. The gastric mucosa was lifted with a solution of saline and methylene blue and ablated in a circumferential manner using HAPC at a power setting of 50 W (Figure 10). 

**Figure 11:**
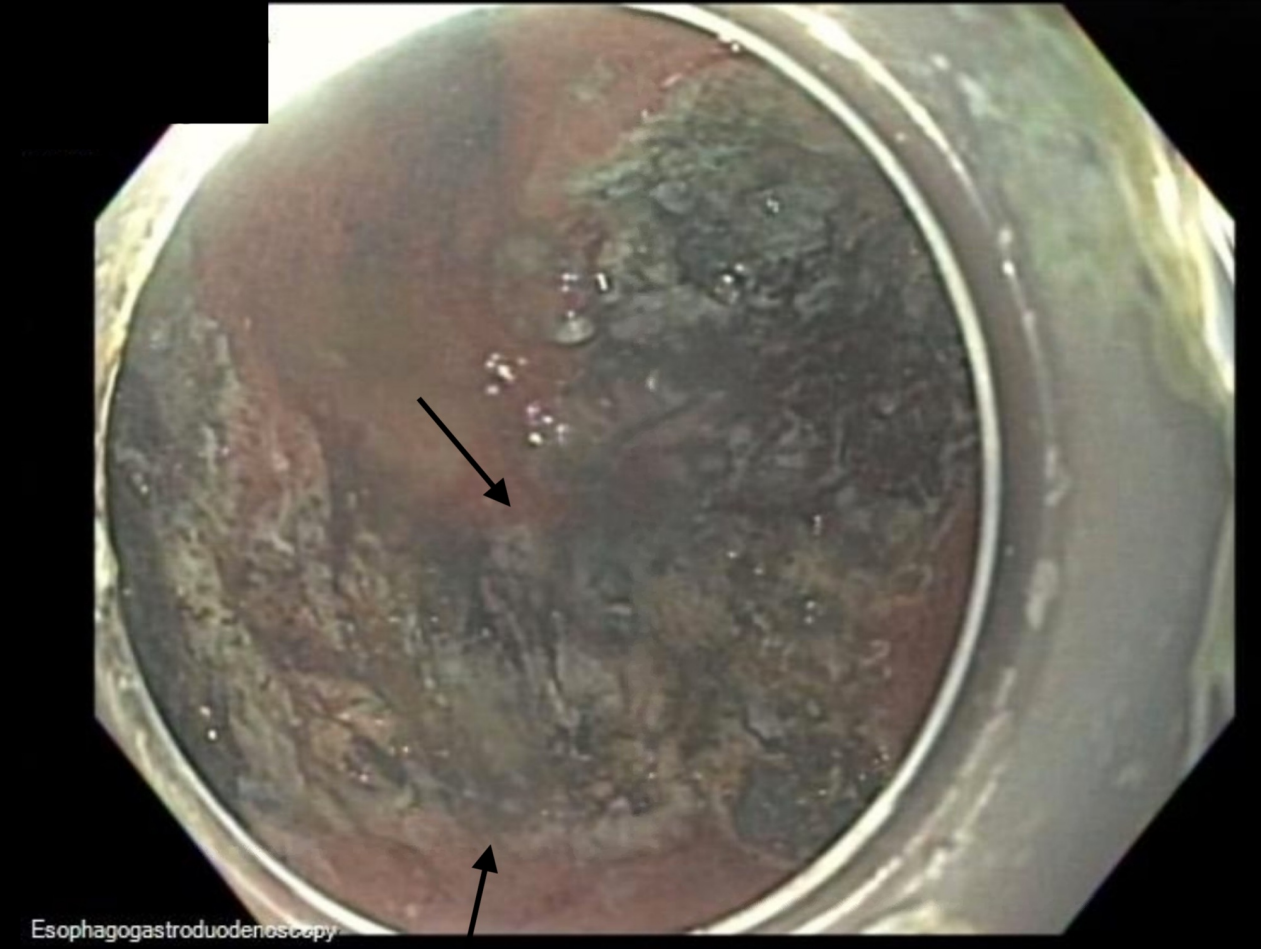
Endoscopic image Endoscopic image showing status post-hybrid argon plasma coagulation.

HAPC was applied in a circumferential manner for comprehensive mucosal coagulation extending from the ESD scar to a 2-cm circumferential margin (Figure 11).

## Discussion

Standard APC employs ionized argon gas to facilitate thermal coagulation [13,16]. There are three APC modes: forced APC, which provides a continuous energy output; pulsed APC, which allows customization of energy output and a number of pulses, pause intervals while maintaining a constant voltage; and precise APC, which provides continuous energy through an increased plasma intensity [13]. APC can be complicated by strictures, perforation, incomplete ablation of the target lesion, and bleeding [13,16,17]. HAPC was first described in 2017. It is a modification of standard APC that combines the application of APC with the ability to create a submucosal injection through the same catheter. It was initially developed for the ablation of refractory Barrett’s mucosa and is now being used for the ablation of residual dysplastic or neoplastic tissue following endoscopic resection [13,14].

**Figure 12:**
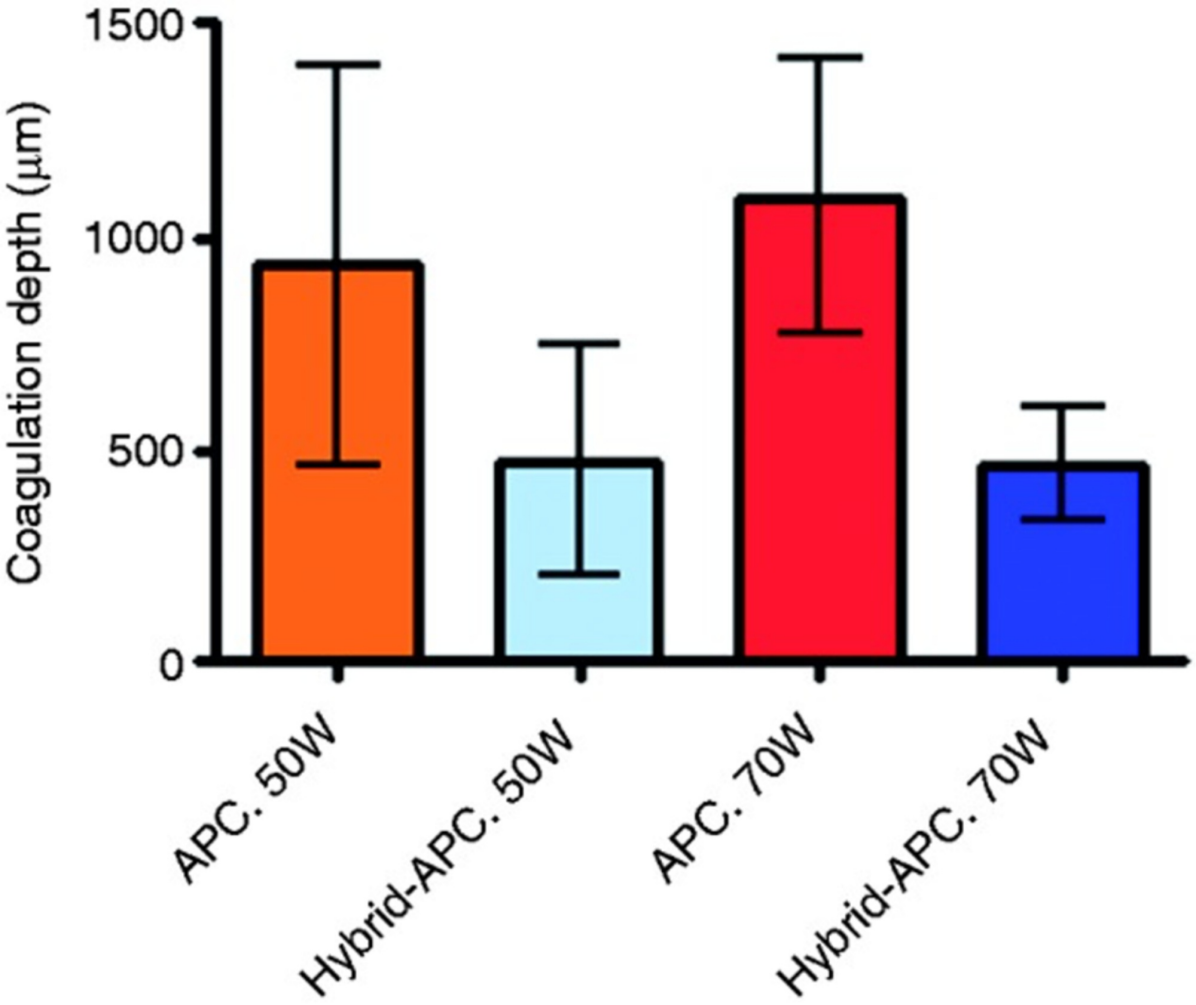
Histologic quantification of the coagulation depth Histologically determined coagulation depth for standard APC and hybrid-APC [18]. APC, argon plasma coagulation.

HAPC improves the complication profile of conventional APC by decreasing thermal injury to deep tissue planes, which may otherwise have resulted in perforation of the muscularis propria [18]. Notably, it does so while maintaining efficacy. Manner et al. reported a series of 50 patients with Barrett’s esophagus who were treated with HAPC. Approximately 96% of the patients achieved macroscopically complete remission from Barrett’s mucosa after a median of 3.5 APC sessions and 85% of them achieved complete histological remission [19]. An ex vivo randomized trial compared standard APC and HAPC, and the coagulation depth was reduced by half when using HAPC [18]. On histological quantification, the coagulation depth of standard APC was 937 ± 469 µm using 50 W and 1,096 ± 320 µm using 70 W. The HAPC depth of coagulation was 477 ± 271 µm using 50 W and 468 ± 136 µm using 70 W (Figure 12).

**Figure 13:**
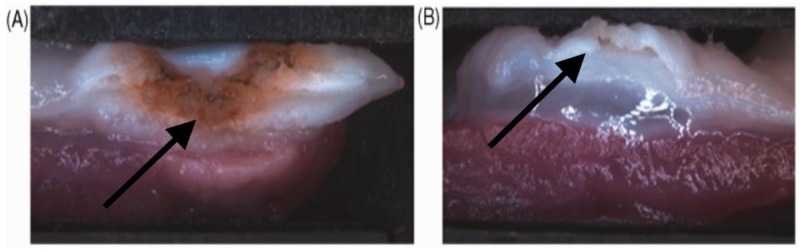
Macroscopically observed damage after standard APC and hybrid-APC In standard APC (A), the damage to the muscle layer can be observed macroscopically. However, with hybrid-APC (B), there is no thermal damage to the muscle layer observed after submucosal cushioning [18]. APC, argon plasma coagulation.

Importantly, this study showed that the muscle layer sustained an injury in the standard APC group, but not in the HAPC group (Figure 13 and Table 1) [18]. 

**Table 1:** Macroscopically measured coagulation depths Macroscopically measured coagulation depths after standard and hybrid-APC [18}. APC, argon plasma coagulation.

Mean ± SD (mm)			
Wattage (W)	Standard APC	Hybrid-APC	P value
50	3.4 ± 0.6	1.6 ± 0.3	0.0006
70	4.4 ± 0.4	2.2 ± 1.1	<0.0001

The decreased overall depth of thermal injury may correlate with a lower rate of complications, specifically stricture formation and perforation. Fujishiro et al. reported that HAPC-associated tissue damage was not observed beyond the submucosal layer, regardless of time duration in their ex vivo study [20]. HybridAPC® is a promising technological advancement providing an efficacious, efficient, and safe addition to the armamentarium of endoscopic tools. Its use in mucosal lesions of the stomach, such as intestinal metaplasia with dysplasia, can be considered based on the clinical scenario.

## Conclusions

HAPC is an effective and safe methodology for ablation widespread gastric intestinal metaplasia with dysplasia.
